# Development of an extensive workflow for comprehensive clinical pharmacogenomic profiling: lessons from a pilot study on 100 whole exome sequencing data

**DOI:** 10.1038/s41397-022-00286-4

**Published:** 2022-08-13

**Authors:** Alireza Tafazoli, Maaike van der Lee, Jesse J. Swen, Anna Zeller, Natalia Wawrusiewicz-Kurylonek, Hailiang Mei, Ruben H. P. Vorderman, Krzysztof Konopko, Andrzej Zankiewicz, Wojciech Miltyk

**Affiliations:** 1 Department of Analysis and Bioanalysis of Medicines, Faculty of Pharmacy with the Division of Laboratory Medicine, Medical University of Bialystok, 15-089 Bialystok, Poland; 2 Clinical Research Centre, University Hospital of Bialystok, 15-276 Bialystok, Poland; 3 Department of Clinical Pharmacy and Toxicology, Leiden University Medical Center, 2333 ZA Leiden, The Netherlands; 4 Department of Clinical Genetics, Medical University of Białystok, 15-089 Białystok, Poland; 5 Sequencing Analysis Support Core, Department of Biomedical Data Sciences, Leiden University Medical Center, 2333 ZA Leiden, The Netherlands; 6 Department of Photonics, Electronics, and Lighting Technology, Faculty of Electrical Engineering, Bialystok University of Technology, 15-351 Bialystok, Poland

**Keywords:** Predictive medicine, Risk factors

## Abstract

This pilot study is aimed at implementing an approach for comprehensive clinical pharmacogenomics (PGx) profiling. Fifty patients with cardiovascular diseases and 50 healthy individuals underwent whole-exome sequencing. Data on 1800 PGx genes were extracted and analyzed through deep filtration separately. Theoretical drug induced phenoconversion was assessed for the patients, using *sequence2script*. In total, 4539 rare variants (including 115 damaging non-synonymous) were identified. Four publicly available PGx bioinformatics algorithms to assign PGx haplotypes were applied to nine selected very important pharmacogenes (VIP) and revealed a 45–70% concordance rate. To ensure availability of the results at point-of-care, actionable variants were stored in a web-hosted database and PGx-cards were developed for quick access and handed to the study subjects. While a comprehensive clinical PGx profile could be successfully extracted from WES data, available tools to interpret these data demonstrated inconsistencies that complicate clinical application.

## Introduction

Pharmacogenomics (PGx) is aimed at reducing adverse drug reactions (ADRs) and lack of efficacy by adjusting drug therapy based on an individual’s genetic profile. Many single gene-drug interactions have been described so far. For interactions with the highest evidence, guidelines and recommendations are available [1]. Not only patients, but also healthy individuals may benefit from PGx testing for future prescriptions by saving the PGx data in electronic health records (EHR) [2]. Currently, targeted genotyping is standard practice in most PGx laboratories for the identification of important variants in the pharmacogenes. However, these panel-based tests are not able to identify rare genomic variants which are expected to have a substantial impact on a patient’s drug response. Sequencing-based methods, on the other hand, are capable of detecting most of the rare variants [3, 4]. These additional variants may help to better explain and predict drug-related phenotypes. Several groups have investigated the utility of next-generation sequencing (NGS) for PGx, both with the use of whole-exome sequencing (WES) as well as whole-genome sequencing (WGS) [5–11]. Such studies for example, demonstrated the utilization of WGS for the identification of putatively functional variants within well-known pharmacogenes. The result successfully represented the missing causative variants underlying drug response phenotypes [12]. However, state-of-the-art high throughput sequencing approaches result in a large amount of data, making it necessary to develop more powerful PGx-bioinformatics tools as well as assess the clinical validity and utility of sequencing-based tests [13]. Multiple tools have been developed and tested in NGS-based PGx studies [14, 15]. We provided a comprehensive review of such tools and their functional algorithms previously [16]. The performance of available haplotyping tools was also compared for *CYP2D6* before. The study showed that while the overall performance was good, there were discrepancies between the individual tools. Nevertheless, a comparison of the utility of these tools for clinical PGx samples and a wide range of genes is yet to be made [17]. In this pilot study, we aim to develop an approach for comprehensive clinical PGx profiling of 100 participants. Also, we introduce a method of deep filtration for dealing with variants in less-studied drug-related genes.

## Methods

### Sample collection

Blood samples from 100 participants of a local and longitudinal observational biomedical project were obtained (50 cardiovascular patients with pulmonary hypertension and ischemic disease and 50 healthy individuals with common demographic features as the control group). The project was approved by the Medical University of Bialystok bioethics committee (approval code: R-I-002/630/2018) and all participants provided informed consent.

### Whole-exome sequencing and primary analysis of data

DNA extraction, NGS library preparation, and quality assessment were performed according to standard manufacturer protocols (Supplementary Material). Pre-Capture Pooling Human All Exon V7” was used. The SureSelect^XT^ kits provide a target enrichment system for Illumina paired-end multiplexed sequencing library preparation. Sequencing was performed using the Illumina NovaSeq 6000 instrument. A standard bioinformatics analysis pipeline for raw data was employed for both GRCh37 and GRCh38 genome builds (Supplementary Material). In short, low-quality bases (read depth <10) were omitted and reads were aligned to the reference genome with the BWA-mem (Burrows-Wheeler) algorithm.

### Data filtration and functional assessment

For variant interpretation, annotation, and initial functional assessment *SnpEff* and *ENSEMBL-VEP* were used [18, 19]. For the PGx assessment, a list of 1800 drug-related genes was prepared. These genes include metabolizer enzymes, drug transporters, drug receptors, and drug-target molecules. They were collected from the *PharmGKB* comprehensive gene list (only genes with at least one annotated variant extracted) (*n* = 1707), all *CPIC* gene-drug lists (*n* = 119), and the FDA table of “Pharmacogenomic Biomarkers” in drug labeling (*n* = 132), plus a systematic search in PubMed for any unannotated but newly introduced drug-related genes (*n* = 17). The relevant keywords selected and specified period applied for choosing state-of-the-art articles. Both the abstract and main text were evaluated systematically and the final result was added to our comprehensive gene list (Supplementary Material). Duplicates were then removed and variant call format (VCF) files were filtered to contain only these 1800 genes’ regions. Variants within these regions were flagged if they were predicted as pathogenic or likely pathogenic by *SnpEff* and *VEP* and went through multiple in silico prediction tools including *VarSeq* (Golen Helix™), *Ensembl* variant table, *gnomAD*, and *ExAC* report on selected variants, *Varsome*, and *VarAFT*. Also, *SWISS-MODEL* [20] and PyMol 2.4 [21] were applied to selected variants (predicted as highly damaging) for the implementation of homology modeling for confirmation of the negative effects of amino acid changes in the related protein.

### PGx analysis with multiple dedicated bioinformatics tools

For analysis of variants in known and well-established PGx genes we used four PGx-dedicated tools: *Stargazer* (V.1.0.8), *Aldy* (V.3.3), *PharmCAT* (V.0.8.0), and *PharmaKU* which uses *Stargazer* V1.2.2 [22–26]. For *Aldy*, *PharmCAT*, and *PharmaKU*, GRCh38 BAM and VCF files were used and the tools were run according to their instructions in the accompanying documentation. *Stargazer* only works with GRCh37, hence the GRCh37-based VCF-only mode was used according to the documentation. All VCFs used in this section were the original files from the first standard analysis steps for NGS output containing all genes without any pre-filtering. Hence, results were obtained from all genes included in the tools which differed between tools. However, core pharmacogenes (defined as actionable in PGx guideline providers) were covered by all.

### Haplotype/diplotype evaluation for well-known pharmacogenes

First, we made a comparison table for results from selected PGx-bioinformatics tools for nine core pharmacogenes: *CYP2B6, CYP2C19, CYP2C9, CYP2D6, CYP3A5, DPYD, SLCO1B1, UGT1A1*, and *VKORC1*. These genes are all present in guidelines from *PharmGKB*, *CPIC*, and *DPWG* in addition to being annotated in *PharmVar*. Results differed depending on the algorithm and variants used by each tool. A “3 vs. 1” conflict rule was used: if the same diplotype and phenotype were called by 3 out of the 4 tools, that was considered the correct assignment. If there was no majority agreement or if one tool did not give any calls, randomly selected discrepancies (to a total of 20 discrepancies) were manually investigated with the use of *PharmVar* to assess what the correct assignment was. The outcome resulted in the conclusion that *Stargazer* was most often correct. Hence, for discrepancies, the *Stargazer* assignment was selected as the correct call.

**Fig. 1:**
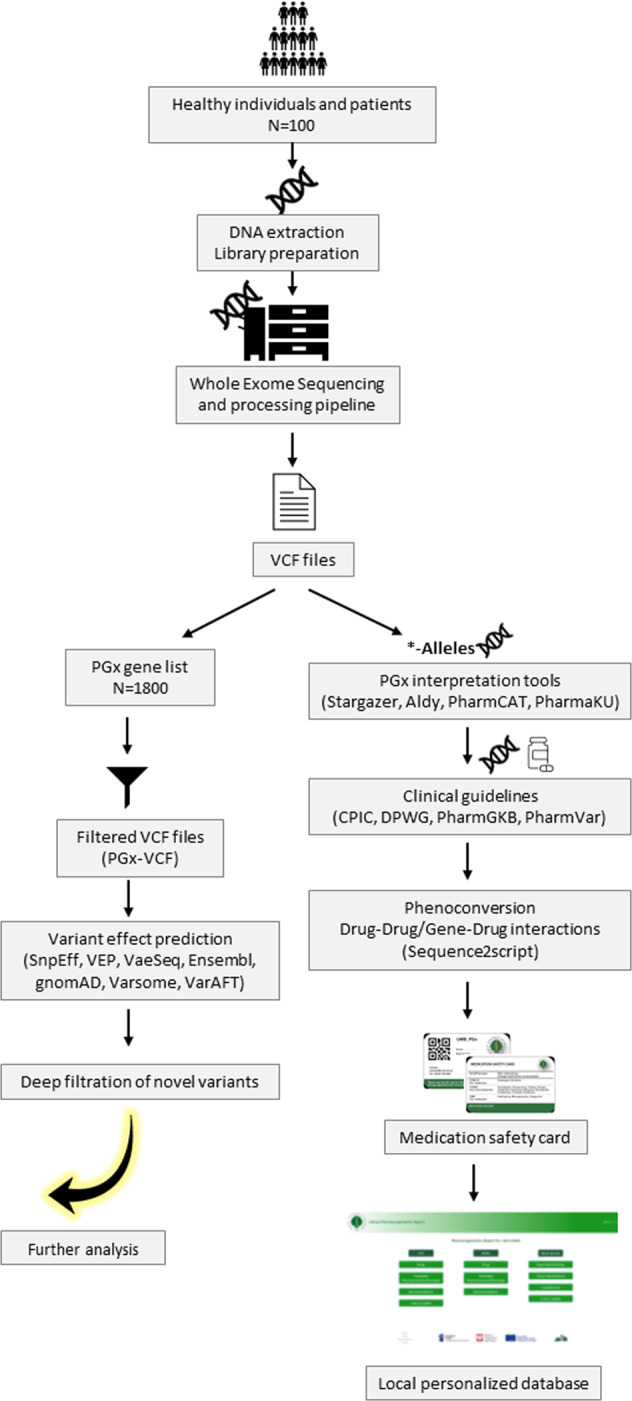
Designed workflow for NGS-based comprehensive clinical PGx test data analysis. Obtained data from WES primary analysis divided into two main categories of variants from less-studied (not interpreted) and well-known pharmacogenes. For the less-studied gene, VCF files were filtered, using the Bed file for 1800 drug-related genes and undergone through deep computational functional assessment and variants effect prediction. Four PGx-dedicated bioinformatics tools are employed for Known genes star allele calling. Clinical guidelines were collected for identified markers in the previous step and samples’ genotype and predicted phenotype were used in *sequence2script* platform for phenoconversion evaluations. Related PGx card created and actionable pharmacovariants for each participant stored in a secure database alongside recommendations from *CPIC* and *DPWG* plus information on novel variants. NGS next-generation sequencing, PGx pharmacogenomics, CPIC Clinical Pharmacogenetics Implementation Consortium, DPWG Dutch Pharmacogenomics Working Group.

Possible drug-drug-gene interactions based on the final predicted metabolizer phenotypes or star alleles were theoretically assessed for all patients. For this, the registered demographic data and complete history of drugs plus clinical manifestations in the case of patients with reported ADRs’ phenotype were used. Possible mismatches between the individuals’ genotype-based prediction of drug metabolism and the true capacity to metabolize drugs were identified by freely available resources as a model of phenoconversion assessment for high throughput DNA sequencing data. Figure 1 illustrates our complete workflow for NGS-based clinical PGx tests for individuals.

### Electronic health records and data storage

**Fig. 2:**
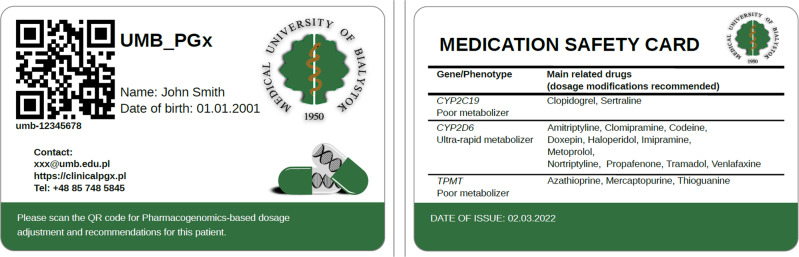
Designed medication safety card for reaching out to individuals’ clinical PGx test result. The card includes both unique number (for the physicians and pharmacists who do not access to QR reader) and QR code which are linked to the secure database (https://clinicalpgx.pl) for each person’s PGx data. Core pharmacogenes with actionable variants for card holder listed in front of the card along with the main substrate which is needed to be considered while prescription for the person. PGx pharmacogenomics.

**Fig. 3:**
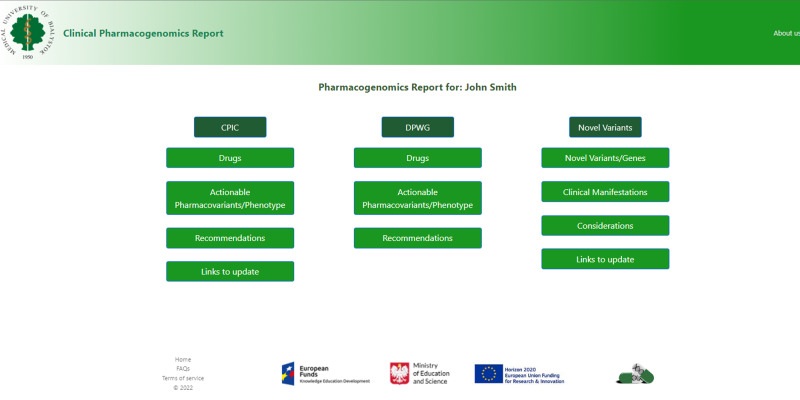
Specific secure personalized database for individuals’ clinical PGx test results and the recommendations from both *CPIC* and *DPWG* plus information on novel identified variants in drug-related genes. See the text for more details. PGx pharmacogenomics, CPIC Clinical Pharmacogenetics Implementation Consortium, DPWG Dutch Pharmacogenomics Working Group.

Haplotypes and phenotypes assigned in the previous step are included in the EHR in university hospital in Bialystok to guide future drug therapy for all participants. Additionally, results are reported back to the participants using a special PGx card as well. Such reporting methods are to allow the utilization of the information based on the provided guidelines or recommendations by *CPIC*, *FDA*, *DPWG*, or other guidelines. Each participant’s profile in the current study includes specific records with information related to *CPIC* and *DPWG* guidelines plus novel variant data in less-known drug-related genes. Access to this database is provided through a publicly available Internet webpage, entitled “clinicalpgx.pl.” (Figs. 2 and 3).

## Results

### WES data analysis for clinical PGx practice

**Fig. 4:**
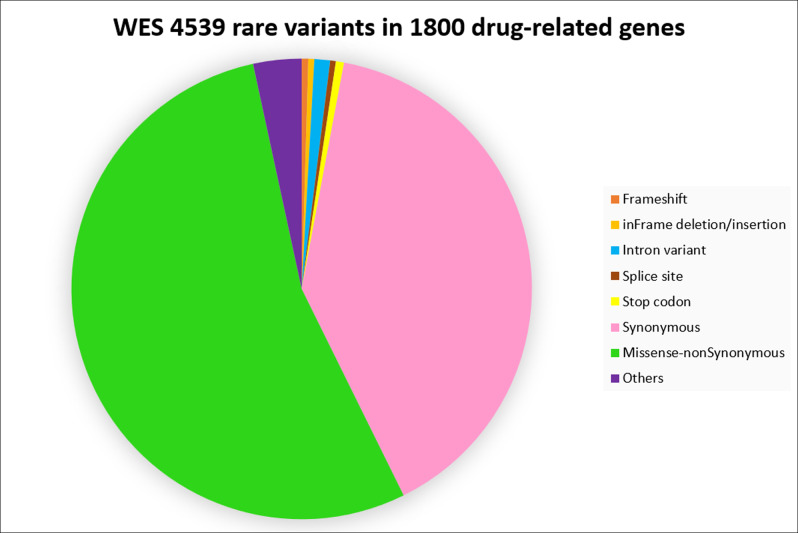
Total rare variants in WES result for all samples. The distribution and functional impact of all rare variants in PGx-VCF files, which contain only drug-related genes. These variants went for the deep computational analysis, mostly for less-studied (not interpreted) drug-related genes. WES whole-exome sequencing, PGx pharmacogenomics.

WES resulted in 1026.75 Gb of 101 bp paired-end reads the output and 93.92% of reads with *Q* > 30. On average, 30,000 variants were identified for each sample. PGx-VCF files displayed between 3300–3600 identified variants in 1800 drug-related genes for each sample. In total 299,297 unique variants from all samples passed the genotype quality and desired read depth (DP) filtrations. Out of the 299,297 variants, 4539 (1.51%) were identified as rare variants with minor allele frequency ≤ 0.01 based on data from the *1K genomes*, *gnomAD*, and *ExAC* databases. Also, the approach revealed 36 variants within our nine core pharmacogenes, with 28 of them considered rare and/or extremely rare in *1K genomes* and *gnomAD*. These 36 variants were not in *CPIC* or *PharmGKB*. Overall, of the 4539 rare variants, there were 21 frameshift, 19 in-frame deletion/insertion, 50 intronic, 18 splice-site, 26 stop codon, 1804 synonymous, and 2447 missense non-synonymous variants in coding regions plus 154 other types of changes (i.e., 27 UTR, 9 initial codon variants, etc.) found (Fig. 4). Multiple functional assessment algorithms identified 115 of the non-synonymous rare variants as damaging. The final step integrated with in silico analysis methods like extra deep filtration, deep computational analysis, and machine learning approaches alongside protein modeling implementation to inferring and providing higher accuracy rate in functional predictions for variants, particularly in less-known drug-related genes. Next, we checked the ability of common genetic bioinformatics tools (*SIFT*, *Polyphen2*, *FATHMM*, *Mutation taster*, *Mutation Assessor, and CAAD*) to identify known impactful variants in pharmacogenes. The evaluation analyzed a list of selected 39 interpreted variants (based on the U-PGx [27] consortiums panel which were previously analyzed through our other investigations and confirmed in PharmVAR) from 11 core pharmacogenes (*CYP2B6*, *CYP2C19*, *CYP2C9*, *CYP2D6*, *CYP3A5*, *DPYD*, *F5L*, *SLCO1B1*, *TPMT*, *UGT1A1*, and *VKORC1*). The results showed that most of the bioinformatics tools used were not successful in identifying these variants as potentially deleterious or impactful. This was particularly evident in the variants associated with a decrease or increase in function. However, variants that are known to be completely deleterious (loss of function) in PGx were identified in half of the cases. This was considered while we used *ExAC-LOF* as one of the main filtration tools for the detection of rare variants in our study. The tool contains information on loss of function variants from *ExAC*, which was one of our main databases for highlighting rare variants as well.

As the common bioinformatics tools like *SIFT*, *Polyphen2*, *CAAD*, etc. were shown to be not suitable for the identification of impactful PGx variants, we may do the pre-filtration of WES data for obtaining only PGx-related genes VCF file and use that in common tools. In this case, all the identified malfunction variants (for example loss of function, which are mostly highlighted by common tools) come from drug-related genes making the downstream processing and detailed analysis more manageable. Then we can go further and do more in silico analysis by other available tools as the confirmation approach for predicted functional assessments.

### Bioinformatics tools for NGS-PGx outcome (comparison of tools result)

**Fig. 5:**
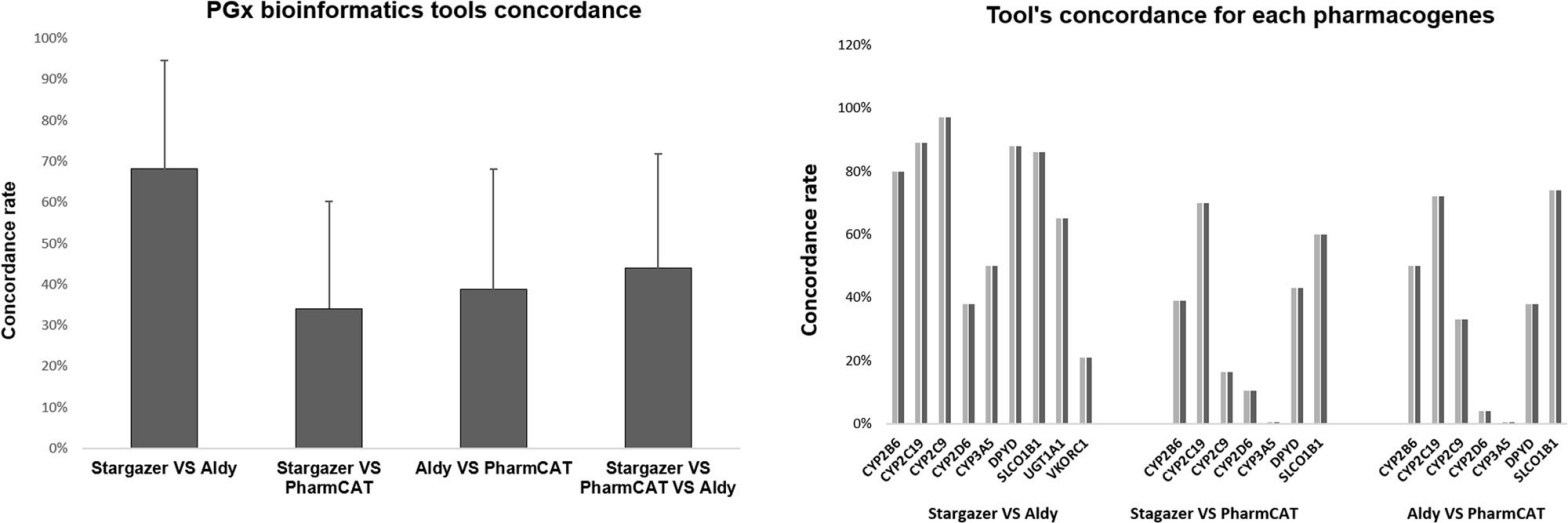
PGx-dedicated bioinformatics algorithms concordance rate for selected nine core pharmacogenes in the current study. The result for the total comparison of tools is illustrated as well. The concordance rate was calculated when there was at least one call for the variants. *PharmaKU* is not included here as it uses *Stargazer* as a built-in algorithm and the result was mainly the same when the correct input data was used (see the main text for more details). *PharmCAT* did not call any alleles for *UGT1A1* and *VKORC1*. PGx pharmacogenomics.

Besides the prediction of yet unused variants in pharmacogenes, we have also explored the results from PGx-dedicated tools for the assignment of *-haplotypes based on well-known pharmacovariants. We evaluated these results in detail and evaluate the potential use of such tools for clinical practice. Four different software tools were used for assigning diplotypes (star alleles) and phenotypes. However, the result from these bioinformatics tools showed discrepancies. While *Aldy* and *Stargazer* showed the most similarity for nine core pharmacogenes, *PharmCAT* was unable to call star alleles for every gene. *PharmaKU* uses *Stargazer* as its basis (v V1.2.2) and accepts genome version GRCh38 as well [26]. While the outcome was mostly concordant with *Stargazer*, there were discrepancies (one per ten calls) mainly due to default calls in *PharmaKU*, especially in the absence of input data. If data on a gene or its variants was missing, *Stargazer* would not provide any results, *PharmaKU* on the other hand would assume the genes were entire wildtype and calls a *1/*1 haplotype. Figure 5 displays the concordance rate for PGx-dedicated bioinformatics tools and for each selected pharmacogene in detail. Also, Table 1 displays multi-tools’ discrepancies reports and provides more details on calling star alleles. The overall result brought some important insights for these tool’s functions, which are worth considering while using such tools in clinical PGx tests: (1) most common cause of discrepancies comes from differences in the variants each tool uses. Also, not all the tools implement phasing for haplotype detection. For example, while *Stargazer* uses Beagle as a built-in algorithm for running the phasing for the samples, *PharmCAT* works best with a phased VCF file as input data. *Aldy* uses only unphased data. (2) the genotype and phenotype assignments are not the same in every tool. For example, *Aldy’s* variant to haplotype translation does not always match PharmVar (i.e., CYP2B6*4 may call as *1). *PharmaKU*, on the other hand, does not provide any information on variants as a web-based report with only star alleles and predicted phenotype. Regarding phenotype assignments, *Stargazer’s* phenotype predictions do not always match the guidelines (e.g., CYP2B6*1/*6 is translated as a normal metabolizer instead of intermediate). Additionally, *Aldy* does not provide phenotype translation in the result, while the other evaluated tools have that. Finally, (3) the transparency and ease of use are different. Differences occur in all aspects of these tools: for example, the necessity for pre-processing of the input data (*Aldy*, *PharmCAT*, and complete mode for *Stargazer*), the comprehensiveness of the report, the technical features, and the genotype and phenotype translation.

**Table. 1:** Multi PGx-dedicated bioinformatics tools’ discrepancies report for each core pharmacogene in current study and the total measurement for such conflicts in 100 WES data.

Genes	*CYP2B6*	*CYP2C19*	*CYP2C9*	*CYP2D6*	*CYP3A5*	*DPYD*	*SLCO1B1*	*UGT1A1*	*VKORC1*
Same result in all tools	70	76	98	8	23	91	43	63	100
3 vs. 1*	15	11	0	1	1	0	45	0	0
2 vs. 2	4	2	1	0	0	0	7	0	0
2 vs. 1 vs. 1	5	2	0	12	0	0	4	0	0
2 vs. 1 (One tool did not call any diplotype)	6	9	1	40	75	6	0	19	Not applicable
All different	0	0	0	39	1	3	1	18	Not applicable
Total conflicts without 3 vs. 1	15	13	2	91	76^b^	9	12	37	–

*Only “3 vs. 1” was not checked for further evaluations. No matched phenotype was removed from the final report. Tools’ report files for the rest of the “vs.” situations are checked manually against *PharmVAR* and *PharmGKB*. Wrong or non-clear calls are interpreted as not accepted calls and removed. The overall concordance rate for all tools: 71% (including 3 vs. 1 scenario) see the main text for more details.

^a^*Stargazer* VCF only mode for calling stars in *CYP2D6* as a highly structural polymorphic gene is not preferred.

^b^*CYP3A5* alleles are defined in a different way in *Stargazer* and *PharmaKU*.

^c^Tools in *SLCO1B1* (less), *UGT1A1*, and *VKORC1* use different allele nomenclature. Hence, the major discrepancies came from different allele names.

### Actionable pharmacovariants in individuals

**Fig. 6:**
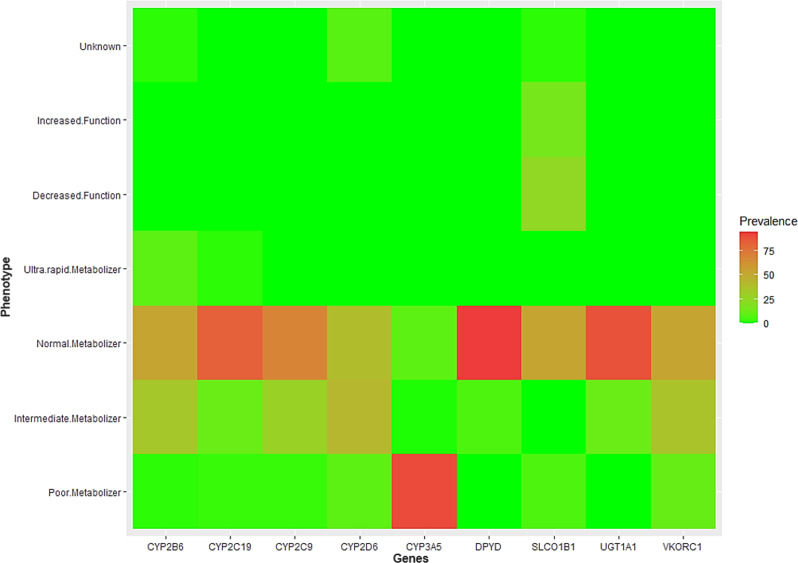
Different metabolizer for nine core pharmacogenes. Distribution and prevalence of different metabolizer phenotypes and related alleles for selected genes in the current study.

The most “non-normal” phenotypes were identified for *CYP2D6*. *CYP3A5*, on the other hand, was the most consistent with almost all samples having a poor metabolizer phenotype. The overall frequency of abnormal alleles leads to aberrant phenotypes within our participants for nine core pharmacogenes were as follow: *CYP2B6* (47%), *CYP2C19* (17%), *CYP2C9* (31%), *CYP2D6* (60%), *CYP3A5* (90%), *DPYD* (6%), *SLCO1B1* (47%), *UGT1A1* (18%), and *VKORC1* (47%). Figure 6 indicates the frequency for each allele in selected genes and linked phenotypes in detail. Moreover, for running the theoretically phenoconversion measurements, particularly drug-drug-gene interactions on a large number of samples, we used *Sequence2script* [28] to identify any potential drug-drug-gene interactions. The assessment, however, showed almost no changes in drug response phenotypes and dosage modifications for our samples. Finally, all phenotypes are included on the PGx result card and on the website (clinicalpgx.pl), which contains both *CPIC* and *DPWG* guidelines to allow them to be accessed by the participants and their healthcare providers. An example of the result from our approach for actionable pharmacovariants and prescription recommendations is provided on “clinicalpgx.pl/data” for anonymous person.

## Discussion

The result of our study on PGx profiling from WES data may be helpful for utilization of bioinformatics tools, specifically PGx-dedicated algorithms, in daily clinical practice. Our investigation also displayed the advantage of pre-filtration of VCF files for only drug-related genes in order to help for identification of more pharmacovariants within such genes. We demonstrated higher accuracy of PGx independent bioinformatics tools, particularly in clinical research, compared to web-hosted algorithms like *PharmaKU* as the tool could be used just as a confirmation for *Stargazer* result, where the input data provided correctly. While the web-based PGx haplotype tools are easier to use, they also seem to be less accurate than the more transparent command line-based programs. In order to touch on the advantages of more in-depth methods in clinical reports, the selection and utilization of correct PGx-dedicated bioinformatics tool(s) must be considered by test centers as well. True applications of PGx bioinformatics algorithms in clinics will bring several advantages, not only in biomarker identification but also in physicians’ accurate decision-making and drug stratification [29]. Choosing the right tool and annotation databases in addition to setting up a consistent workflow for routine practice in clinical centers requires advanced knowledge and awareness of existing tools or resources and their functional approaches in variant interpretations [30].

Even though all the available PGx-dedicated bioinformatics tools are limited to the specific number of pharmacogenes and included the distinct number of pharmacovariants in their panel, most of these curated genetic variations come with clinical guidelines and annotations for treatment modifications as well. Hence, applying multi-tools for including more genes and variants seems reasonable. So far, most studies reported the advantages of using PGx bioinformatics tools in clinical investigations but as a separate entity [31, 32]. For those reported the multi-tool utilization, again not all of the genes in all samples were evaluated in that way [33]. Among PGx-dedicated bioinformatics algorithms, we propose to use at least two of such tools for providing more confident haplotype calls. However, an important limitation of applying different tools would be the necessity for running the alignment part for different reference genomes as some of them might need GRCh37 while the others work with GRCh38. In our study, we tried to use NCBI’s genome remapping service (https://www.ncbi.nlm.nih.gov/genome/tools/remap) to perform this re-alignment and liftover from GRCh38 to GRCh37. However, after evaluating of results, we noticed that the approach led to the exclusion of some important PGx variants. Therefore, we decided to perform a separate realignment with GRCh37 assembly as the reference for the raw data. These data were subsequently used by *Stargazer*.

The outcome of our result in the adaptation of PGx-dedicated bioinformatics tools for clinical interpretation of PGx variants may help clinicians to improve the implementation of the NGS-guided clinical PGx tests. Once the utilization of such computational assessments is established in the center, the related healthcare system may benefit from the fast and more accurate PGx marker diagnosis in a shorter turnaround time.

Also, it is worth considering that not all types of PGx variants may be identified by common bioinformatics tools. As we have shown, increased and decreased functions (rapid and intermediate metabolizers) are mostly ignored by tools like *SIFT*, *Polyphen2*, *FATHMM*, *Mutation taster*, *Mutation Assessor*, etc. Therefore, it might be valuable to filter PGx regions from VCF files to select candidates for in silico validation studies as opposed to using inaccurate in silico tools for the assessment of variant impact. This type of approach would be for novel variants with unknown significance, which have an impact on the protein (e.g., missense or frameshift variants) [34]. Nevertheless, the workflow in this level for the current study brought many interesting outcomes for novel and/or not-annotated variants in our samples, which the interpretation and further analysis are still in progress. Today, computational assessments proved to be a promising approach for the translation of novel variants into healthcare [35, 36].

Besides gene-drug interactions it is also of importance to consider phenoconversion for improving accuracy rate for phenotype prediction in personalized therapy area too. Under the influence of comedication the activity of an enzyme can switch, for example from intermediate to normal metabolizer due to an inducer effect. For instance, proton-pump inhibitors can reduce CYP2C19 activity and thereby convert a normal metabolizer phenotype to an intermediate metabolizer phenotype. This can, in turn, has an impact on other drugs used by the patients. The use of both proton-pump inhibitors (CYP2C19 inhibitor) and clopidogrel (CYP2C19 substrate) is highly likely in a cardiovascular cohort such as ours, therefore it is important to be aware of these types of drug-drug-gene interactions. Future research and investigations will need to take comedication into account when studying the impact of PGx on clinical outcomes.

Integration of computational assessment and bioinformatic functional analysis of pharmacovariants within high throughput DNA sequencing data is rapidly expanding. However, while a comprehensive clinical PGx profile could be successfully extracted from WES data, available tools to interpret such data are not consistent for all pharmacogenes and show several discrepancies compared to each other. Moreover, WES data demonstrates an abundance of variants not yet used in clinical practice. To bring the translation of such technologies into daily clinical setting the clinical validity and utility of dedicated bioinformatics tools should be investigated more.

## Supplementary information

Supplementary material

## Data Availability

The data are available from the corresponding author at Department of Analysis and Bioanalysis of Medicines, Medical University of Bialystok on reasonable request.
