# Supplementary material for: Development of an extensive workflow for comprehensive clinical pharmacogenomic profiling: lessons from a pilot study on 100 whole exome sequencing data
Source: Pharmacogenomics J. 2022 Aug 13;22(5-6):276–83. doi: 10.1038/s41397-022-00286-4 (PMC9674517; doi:10.1038/s41397-022-00286-4)
Supplement: Supplementary file 1 — Supplementary material [file 41397_2022_286_MOESM1_ESM.docx]

**Supplementary material 1**

*DNA extraction, NGS library preparation, and quality assessment*

DNA isolation and purification from whole blood sample was performed with the "QIAGEN, QIAamp DNA Blood Mini Kit (50)", based on product manufacturer instruction. Invitrogen™ Qubit™4 and DeNovix™ nanodrop were used for nucleic acid quality control and measuring DNA concentration. Quality of the pooled libraries was assessed with the Qubit™4 and Agilent™ 2200 TapeStation system.

*Whole exome sequencing*

For sequencing the illumina NovaSeq 6000 instrument with S2 flowcell and paired end read mode was used. The average of 172X depth of coverage calculated for WES outcome based on sample size and best practice formula in illumina sequencing coverage calculator (support.illumina.com). However, normally the real depth after sequencing run would be different for various samples (in our case, 100-130X).

*Primary analysis of WES data*

Standard bioinformatics analysis pipeline for raw fastq reads utilized with fastqc and trimmomatic as the initial tools. The alignment part was performed by BWA algorithm. The Picard and GATK toolkit were used to mark duplicates, variant calling and for variant- and base recalibration. HaplotypeCaller was used to call germline SNPs and indels via local re-assembly of haplotypes. Finally, MultiQC was used for visualisation of the data quality and statistics.

*Data filtration and functional assessment*

Alongside the main sources for PGx, our systematic search in pubmed for any unannotated but newly introduced drug-related genes utilized the following procedure: we used the keywords: "Pharmacogenomics genes, Pharmacogenetic gene, drug-related gene, drug metabolizer gene, drug transporter gene, drug target gene, personalized medicine + gene, personalized therapy + gene, individualized therapy + gene" for studies published after 2021. The abstracts were screened to check if the selected keyword expansion were related to PGx context. Finally, full-text article assessed for the genes of direct implication on PGx research. After combining input from all sources, duplicate genes were removed. The resulting list was made into a BED file and the VCF files were filtered on the locations in this BED file using BCFtools V.1.15.1. This PGx-VCF file, was used in next step which was a functional assessment of multi-bioinformatics tools. The workflow used a deep computational analysis to assign predicted effects of the variants in the VCF files.

**Study limitations**

The main limitations for our investigation would be the sample size which may restrict statistical analysis of the result as well and several false negative results for novel variants in drug-related genes where the available common or PGx-dedicated bioinformatics tools may not recognize increase or decrease functions. Replication studies like applying Sanger sequencing also recommended for NGS-based clinical tests. However, this procedure could be ignored if the NGS test result contain high quality data specifically for SNVs and small InDels (doi: 10.1038/s41598-021-85182-w).
